# Adherence to the Vegetable-Fruit-Soy Dietary Pattern, a Reference From Mediterranean Diet, Protects Against Postmenopausal Breast Cancer Among Chinese Women

**DOI:** 10.3389/fnut.2022.800996

**Published:** 2022-03-29

**Authors:** Shang Cao, Linchen Liu, Qianrang Zhu, Zheng Zhu, Jinyi Zhou, Pingmin Wei, Ming Wu

**Affiliations:** ^1^Department of Epidemiology and Health Statistics, Southeast University, Nanjing, China; ^2^Department of Rheumatology, Zhongda Hospital, School of Medicine, Southeast University, Nanjing, China; ^3^Department of Chronic Disease Control, Jiangsu Provincial Center for Disease Control and Prevention, Nanjing, China

**Keywords:** breast cancer, Mediterranean diet, cancer prevention, molecular subtype, vegetable-fruit-soy diet

## Abstract

**Background:**

The diet-center hypothesis has gained much support from the apparent protective effect of the Mediterranean diet on breast cancer. However, the evidence of the association between Mediterranean diet adherence and breast cancer molecular subtypes remains small, especially in non-Mediterranean populations.

**Methods:**

The subjects from the Chinese Wuxi Exposure and Breast Cancer Study, a population-based case-control study, included 818 patients and 935 healthy controls. A validated food frequency questionnaire used for diet assessment and a modified version of the alternate Mediterranean Diet Score, which is called the alternate Chinese Diet Score, was developed to assess adherence to a migrated Chinese version of the Mediterranean diet, which we called the vegetable-fruit-soy dietary pattern. Soy foods, rapeseed oil, and coarse cereals replaced legumes, olive oil, and whole grains reflecting the cuisine of the region. We examined the association between the vegetable-fruit-soy diet adherence and breast cancer risk, stratified by menopause status (pre- or postmenopausal) and receptor status [estrogen-receptor (ER), progesterone-receptor (PR) status, and human epidermal growth factor 2 (HER2)] oncogene expression, followed by five specific combinations (ER+, ER–, ER+/PR+,ER–/PR–, and ER–/PR–/HER2–).

**Results:**

The results suggest that the vegetable-fruit-soy dietary pattern was inversely associated with postmenopausal breast cancer risk [4th vs. 1st quartile, odds ratio (OR) = 0.57, 95%CI = 0.41, 0.80; *P* trend < 0.001] and that the inverse association was somewhat stronger to detect among ER- subtypes (OR = 0.63; 95%CI = 0.37, 0.94; *P* trend = 0.003) and ER–/PR–subtypes (OR = 0.64; 95%CI = 0.41, 0.93; *P* trend = 0.012). We did not observe any significant association between the vegetable-fruit-soy diet characteristics and ER+ subtype, as well as between PR+ and ER+/PR+ subtypes.

**Conclusion:**

The favorable influence from the Mediterranean diet may also apply to Chinese women. The vegetable-fruit-soy dietary pattern may reduce the risk of postmenopausal breast cancer, particularly among ER- subtype, and ER–/PR–subtype.

## Introduction

Some extensive cohort studies reported the inverse association for the dietary pattern characterized by the traditional diet among populations in Mediterranean countries of breast cancer risk (1–8), which implies that the Mediterranean diet may be a unique dietary combination for breast cancer prevention. Review studies are reinforcing this hypothesis (9). The Mediterranean diet is a way of eating based on the traditional cuisines of Greece, Italy, and other countries that border the Mediterranean Sea. Plant-based foods, such as whole grains, vegetables, legumes, fruits, nuts, seeds, herbs, and spices, are the foundation of the diet.

Of particular interest were the low breast cancer incidences that have been reported in Asian countries (10). It has been noticed that the traditional Asian diet has much in common with the Mediterranean diet, as both of them are characterized by the high consumption of vegetables (11) and fruits, take more high-quality white meat rather than red meat (12, 13), and place emphasis on monounsaturated:saturated fat ratio (14). So, a hypothesis is proposed that the health benefits of the Mediterranean diet should be transmissible if the food components and their combinations in the Mediterranean diet play an etiologic role in breast cancer.

However, it is almost impossible to perform a long-term feeding trial in a large-scale population in achieving high compliance to follow the Mediterranean diet except with intensive intervention (15). Therefore, based on the original Cretan Mediterranean diet, we modified the diet according to Chinese diet habits; soy foods, rapeseed oil, and coarse cereals replaced legumes, olive oil, and whole grains, reflecting the cuisine of the region. In addition, due to the differences in etiology, it is important to distinguish between premenopausal and postmenopausal breast cancer and different breast tumor molecular subtypes. The recent meta-analysis summaries of six cohort studies that included 680,450 subjects suggested that the Mediterranean diet adherence (MD-adherence) is inversely associated with postmenopausal breast cancer and the inverse association is somewhat stronger among estrogen-receptor (ER)- tumors than ER+ tumors (16). If MD adherence is significantly associated with ER- tumors, which may have important implications for prevention, because of the poorer prognosis of ER- breast cancer.

This study aims to scrutinize the association between a migrated Chinese version of the Mediterranean diet, which we called the vegetable-fruit-soy dietary pattern, and breast cancer risk among Chinese women, stratified by the menopausal status and different breast tumor molecular subtypes.

## Materials and Methods

### Study Subjects

The Chinese Wuxi Exposure and Breast Cancer Study (2013–2014) was a case-control study of the role of biology, diet, lifestyle, and environmental factors in the etiology of breast cancer in Asian women. Subjects were all adult women and were restricted to local residents who have lived in Wuxi for at least 5 years. All newly diagnosed (the diagnosis date was within 1 year before enrolment) women with breast cancer (ICD code: C50) among local residents identified by cancer registries were eligible to be included as cases. Secondary and recurrent cancers were excluded. Controls were derived from the local area as cases and were 1:1 individually matched with cases by age (±2 years) and residence. As personal information such as name, address, date of birth, and sex for all residents was available in the local demographic information database; eligible controls were randomly identified from this database. For each control chosen, two additional subjects were selected as a backup at the same time. When the first control could not be interviewed, an alternative was enrolled in the study. The selection procedure repeated until an eligible subject was interviewed. A total of 1,042 eligible breast cancer cases and 1,042 health controls were identified during the study period. Case and controls with major dietary habits changed over the past 10 years, which were excluded from this study. In analysis, 818 cases and 935 controls were included, with a frequency match (cases and controls have the same distributions over age and residence). All subjects gave voluntary written informed consent to participate in this research. The Institutional Review Boards of the Jiangsu Centers for Disease Control approved the study protocol.

### Data on Diet

The usual diet was assessed by a validated, semiquantitative food frequency questionnaire (FFQ), which included 149 items along with the recipes commonly used in China; a detailed description is given in Zhao et al. (17). Nutrient and energy intake were calculated through the Chinese Food Composition Database (2018, 6th version).

Dietary intake assessment included whether the food was consumed, consumption frequency (times of per day/week/month/year), and the average amount of food consumption at each time. The 149 food items in the FFQ were classified into 18 predefined food groups based on similarities in nutrient profile and culinary usage.

### Alternate Chinese Diet Score

A modified version on the alternate Mediterranean Diet Score (aMED) which we called the alternate Chinese Diet Score (aCHD) was developed to assess adherence to a migrated Chinese version of the Mediterranean diet, which we called the vegetable-fruit-soy dietary pattern. Soy foods, rapeseed oil, and coarse cereals replaced legumes, olive oil, and whole grains, reflecting the cuisine of the region. The aMED (18, 19) is an adapted version of the traditional Mediterranean Diet Score created by Trichopoulou et al. (20, 21). The aMED established by Fung et al. (18) included nine dietary components that are typical of the Mediterranean diet. For each of the presumed beneficial food items [vegetables, legumes, fruits, nuts, whole grains, fish, and the ratio of monounsaturated to saturated fatty acid intake (MUFA:SFA)], one point was given when the intake was at least the sex-specific median intake, and zero otherwise. For red and processed meat, 1 point was given (and 0 otherwise) when the intake was below the sex-specific median intake. In the full aMED, 1 additional point is normally given when alcohol intake is between 5 and 25 g/day, and 0 otherwise (19), with a 9-point sum score (minimal to maximal conformity). The aCHD also ranged from zero to nine points in the present analysis (Appendix).

To test the role of the vegetable-fruit-soy dietary pattern in the etiology of breast cancer, we design an interaction test between the aCHD and the duration of diet habits. The “duration” indicator was based on interviewing subjects about how long they have maintained their current diet habits, and the medians were calculated based on the distribution of the premenopausal controls and postmenopausal controls, respectively.

### Lifestyle, Anthropometric, Medical History, and Reproductive History Data

Demographic, lifestyle characteristics, menstrual and reproductive events, dietary intake, medical history, and physical activity-related data were obtained from a structured questionnaire through in-person interviews conducted by trained interviewers. Anthropometric measures were obtained by trained personnel following standardized protocols; the core elements of anthropometry are height, weight, body mass index (BMI), body circumferences (waist, hip, and limbs), and skinfold thickness. Physical activity was measured by referencing the Global Physical Activity Questionnaire (22). Women were considered to be postmenopausal at an absence of menstruation in the past 12 months.

## Subtypes of Breast Tumor

The molecular subtypes of breast tumor based on ER status, progesterone-receptor (PR) status, and human epidermal growth factor 2 (HER2) oncogene expression, followed by five specific combinations (ER+, ER–, ER+/PR+, ER–/PR–, and ER–/PR–/HER2–).

### Statistical Analysis

The demographic characteristics and anthropometric measures of the subjects were presented as means± SD for normal distribution or median (Q1 and Q3) for non-normal distribution and frequency percentages. A Student’s *t*-test or the Wilcoxon rank-sum test and chi-squares tests were performed for continuous and categorical variables, respectively, to compare the differences between cases and controls.

To estimate the impact of the separate foods component of the vegetable-fruit-soy dietary pattern on breast cancer, we calculated adjusted odds ratios (ORs) for the intake ≥ median of the specific foods component compared to the intake < median (as reference), medians were calculated based on the distribution of the premenopausal controls and postmenopausal controls, respectively.

Associations between the aCHD score (divided into four quartiles, as a nominal predictor) and breast cancer were estimated in terms of adjusted ORs and respective 95%CIs by logistic regression models.

In the sensitivity analysis, soy foods were often considered to be an independent potential benefit for breast cancer prevention in Asian populations (23, 24). Thus, we examined the independent effect of soy foods on breast cancer, adjusting other foods components that were considered to be beneficial factors in the migrated vegetable-fruit-soy diet, namely, vegetables, fruit, nuts, cereals, fish, and monounsaturates. In addition, we designed a modified soy-free aCHD score with all soy foods in computation excluded for scrutinizing the joint effects of the vegetable-fruit-soy dietary pattern except for soy foods.

In analyses, we controlled potential confounders associated with breast cancer risk, included age at diagnosis for cases or enrollment for controls (by years), education (ordered as illiterate and primary, middle, and high school, university and above), tobacco smoking (no or yes: including smoking and second-hand smoking ≥ 3days/week), moderate physical activity (minutes/day), oral contraceptives use (no or yes: current use or ever use), hormone replacement therapy (no or yes: current use or ever use), family history of breast cancer (no or yes: in a first-degree relative), history of benign breast disease (no or yes: including lactation mastitis, plasma cell mastitis, cyclomastopathy, fibroadenoma of breast, and galactocele), age at menarche (by years), number of full term births (ordered as 0, 1, 2,*or*≥3), age at first full-term delivery (by years), breastfeeding (no or yes), height (by cm), BMI (in kg/m^2^), energy intake (kcal/day). In addition, postmenopausal stratification analysis was further adjusted for the menopausal age (by years). All subjects were included in the present analysis, with the missing covariates data imputed by using the fully conditional specification Multivariate Imputation by the Chained Equations method. Interactions were examined by the likelihood ratio tests (LRTs).

All analyses were performed with R version 4.0.2 (The R Project for Statistical Computing, China).^1^ Using CIs to measure effect size and to gauge precision, and precise *P*-values (not just whether *P*-values are above or below 0.05 or some other threshold), *P*-value near 0.05 taken by itself offers only weak evidence against the null hypothesis.

## Results

The demographic characteristics and anthropometric measures of the subjects stratified by menopausal status are presented in Table 1. The education level of the case group was lower than that of the control group, and the proportion of overweight rate, family history of breast cancer, and history of benign breast disease was higher than that of the control group.

**TABLE 1:** Characteristics of subjects for analysis, stratified by menopausal status.

	All subjects (*n* = 1,753)					Premenopausal (*n* = 600)					Postmenopausal (1,153)				
	Cases = 818		Controls = 935			Cases = 214		Controls = 386			Cases = 604		Controls = 549		
Variable	*n*	%	*n*	%	*P*-value^a^	*n*	%	*n*	%	*P*-value^a^	*n*	%	*n*	%	*P*-value^a^
**Age at enrollment (years)**															
<50	295	36.1%	328	35.1%	0.41	189	88.3%	295	76.4%	0.02	106	17.5%	33	6.0%	<0.01
50–60	239	29.2%	304	32.5%		20	9.3%	81	21.0%		219	36.3%	223	40.6%	
>60	284	34.7%	303	32.4%		5	2.3%	10	2.6%		279	46.2%	293	53.4%	
Median (Q1, Q3)	54	(46, 63)	53	(46, 62)		44	(41, 47)	45	(41, 49)		58	(51, 65)	60	(55, 67)	
**Area**															
Urban	447	54.6%	515	55.1%	0.86	116	54.2%	208	53.9%	0.94	331	54.8%	307	55.9%	0.70
Rural	371	45.4%	420	44.9%		98	45.8%	178	46.1%		273	45.2%	242	44.1%	
**Educational level**															
Illiterate and primary	256	31.3%	228	24.4%	< 0.01	32	15.0%	27	7.0%	< 0.01	224	37.1%	201	36.6%	0.81
Middle and high school	497	60.8%	568	60.7%		146	68.2%	251	64.9%		351	58.0%	317	57.7%	
University and above	65	7.9%	139	14.9%		36	16.8%	108	28.1%		29	4.8%	31	5.6%	
**Smoking**															
No	453	55.4%	541	57.9%	0.30	119	55.6%	210	54.4%	0.78	334	55.3%	331	60.3%	0.09
Yes	365	44.6%	394	42.1%		95	44.4%	176	45.6%		270	44.7%	218	39.7%	
**Moderate physical activity (minutes/day)**															
Median (Q1, Q3)	16.4	(7.0, 21.0)	16.3	(7.0, 21.0)	0.66	10.5	(5.7, 17.8)	10.6	(6.9, 18.7)	0.68	13.9	(7.0, 21.0)	14.0	(7.0, 23.0)	0.08
**Oral contraceptives use**															
No	653	79.8%	767	82.0%	0.24	42	19.6%	68	17.6%	0.54	123	20.4%	100	18.2%	0.36
Yes	165	20.2%	168	18.0%		172	80.4%	318	82.4%		481	79.6%	449	81.8%	
**HRT use**															
No	774	94.6%	899	96.1%	0.13	202	94.4%	368	95.3%	0.61	572	94.7%	531	96.7%	0.09
Yes	44	5.4%	36	3.9%		12	5.6%	18	4.7%		32	5.3%	18	3.3%	
**Family history of breast cancer**															
No	730	89.2%	888	95.0%	< 0.01	187	87.4%	362	93.8%	0.01	543	89.9%	526	95.8%	< 0.01
Yes	88	10.8%	47	5.0%		27	12.6%	24	6.2%		61	10.1%	23	4.2%	
**History of begin disease**															
No	495	60.5%	635	67.9%	< 0.01	98	45.8	212	54.9	0.03	397	65.7%	423	77.0%	< 0.01
Yes	323	39.5%	300	32.1%		116	54.2	174	45.1		207	34.3%	126	23.0%	
**Age at menarche**															
10–14	240	29.3%	281	30.1%	0.93	103	48.1	169	43.8%	0.35	137	22.7%	112	20.4%	0.49
15–16	317	38.8%	355	38.0%		80	37.4	144	37.3%		237	39.2%	211	38.4%	
17–22	261	31.9%	299	32.0%		31	14.5	73	18.9%		230	38.1%	226	41.2%	
Median (Q1, Q3)	16	(14, 17)	16	(16, 17)	0.72	15	(14, 16)	15	(14, 16)	0.19	16	(15, 17)	16	(15, 17)	0.26
**Number of full-term births**															
0	13	1.6%	12	1.3%	0.56	4	1.9%	6	1.6%	0.36	9	1.5%	6	1.1%	0.09
1	467	55.9%	548	58.6%		164	76.6%	318	82.4%		293	48.5%	230	41.9%	
2	253	30.9%	263	28.1%		41	19.2%	53	13.7%		212	35.1%	210	38.3%	
≥3	95	11.6%	112	12.0%		5	2.3%	9	2.3%		90	14.9%	103	18.8%	
**Age at first full-term delivery**															
25	546	66.7%	627	67.1%	0.14	158	73.8%	274	71.0%	0.07	388	64.2%	353	64.3%	0.72
25–29	234	28.6%	281	30.1%		48	22.4%	107	27.7%		186	30.8%	174	31.7%	
>29	38	4.6%	27	2.9%		8	3.7%	5	1.3%		30	5.0%	22	4.0%	
Median (Q1, Q3)	25	(23, 26)	25	(23, 26)	0.85	24	(23, 26)	24	(23, 26)	0.15	25	(23, 26)	25	(23, 26)	0.98
**Breastfeeding**															
No	276	33.7%	287	30.7%	0.17	45	21.0%	63	16.3%	0.15	231	38.2%	224	40.8%	0.38
Yes	542	66.3%	648	69.3%		169	79.0%	323	83.7%		373	61.8%	325	59.2%	
**Height (cm)**															
Median (Q1, Q3)	157	(154, 160)	157	(153, 160)	0.20	159	(155, 162)	158	(155, 162)	0.93	156	(153, 160)	155	(152, 159)	< 0.01
**BMI (kg/m^2^)**															
Underweight (<18.5)	21	2.6%	15	1.6%	< 0.01	10	4.7%	8	2.1%	0.07	11	1.8%	7	1.3%	0.01
Normal (18.5–23.9)	328	40.1%	471	50.4%		104	48.6%	216	56.0%		224	37.1%	255	46.4%	
Overweight (>24)	469	57.3%	449	48.0%		100	46.7%	162	42.0%		369	61.1%	287	52.3%	
Median (Q1, Q3)	24.6	(22.6, 26.8)	23.8	(21.9, 26.1)	< 0.01	23.8	(21.9, 25.9)	23.3	(21.5, 25.7)	0.18	24.9	(22.4, 26.3)	24.1	(22.4, 26.3)	< 0.01
**Energy intake (kcal/day)**															
Median (Q1, Q3)	1635.2	(1347.3, 2018.3)	1667.9	(1374.9, 2032.7)	0.48	1649.5	(1389.9, 1982.9)	1710.4	(1433.9, 2062.3)	0.16	1625.0	(1336.7, 2026.8)	1645.4	(1327.7, 1995.5)	0.64
**Menopause age**															
Median (Q1, Q3)											50	(47, 52)	50	(47, 53)	0.10

*^a^P-values were calculated based on the t-test for normal distribution and the Wilcoxon rank-sum test for non-normal distribution; for frequency distribution, P-values were calculated based on the chi-squared test.*

**FIGURE 1:**
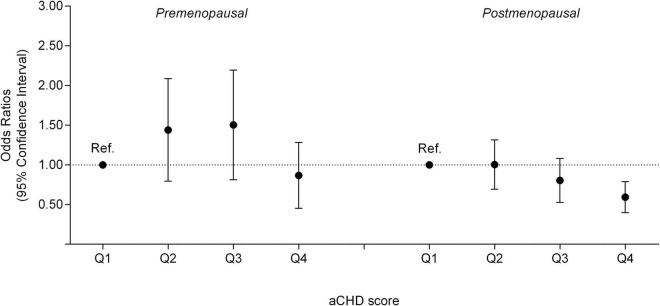
The quartiles of the aCHD with premenopausal and postmenopausal breast cancer risk.

The association between the vegetable-fruit-soy diet adherence and breast cancer is shown in Table 2. We found a significant trend of postmenopausal breast cancer risk reductions with a high aCHD score (*P*-value for trend < 0.001) and women with the highest quartile aCHD score had a significantly lower risk compared with women with the lowest quartile aCHD score (OR = 0.57; 95%CI = 0.41, 0.80), while no significant association was found for premenopausal breast cancer (Figure 1). Furthermore, we found a significant interaction between the vegetable-fruit-soy diet adherence and the duration of diet habits on postmenopausal breast cancer (*P* interaction = 0.013). Long-term (≥median) vegetable-fruit-soy diet adherence (highest quartile of aCHD) significantly reduced postmenopausal breast cancer risk compared with short-term (<median) adherence and showed lowest quartile of aCHD (OR = 0.42, 95%CI = 0.26–0.69), Table 3.

**TABLE 2:** Associations (OR and 95%CIs) between the alternate Chinese Diet Score (aCHD), the soy-free alternate Chinese Diet Score (soy-free aCHD), and soy foods daily intake and breast cancer, stratified by menopausal status.^a^

	All		Premenopausal		Postmenopausal		*P* interaction^g^
Diet	Case/Control	Adjusted OR^b^ (95%CI)	Case/Control	Adjusted OR^b^ (95%CI)	Case/Control	Adjusted OR^b^ (95%CI)	
**aCHD (score)^c^**							
Quartile 1	242/252	1.00 (reference)	52/106	1.00 (reference)	190/146	1.00 (reference)	0.022
Quartile 2	233/228	1.07 (0.83, 1.37)	63/95	1.34 (0.85, 2.13)	170/133	0.97 (0.71, 1.33)	
Quartile 3	193/211	0.95 (0.73, 1.24)	58/83	1.40 (0.87, 2.24)	135/128	0.78 (0.56, 1.09)	
Quartile 4	150/244	0.64 (0.49, 0.84)	41/102	0.80 (0.49, 1.31)	109/142	0.57 (0.41, 0.80)	
*P*-trend		0.002		0.480		< 0.001	
**Soy-free aCHD (score)^d^**							
Quartile 1	349/374	1.00 (reference)	83/157	1.00 (reference)	266/217	1.00 (reference)	0.018
Quartile 2	219/224	1.05 (0.83, 1.33)	63/94	1.24 (0.81, 1.88)	156/130	0.96 (0.71, 1.29)	
Quartile 3	142/177	0.86 (0.66, 1.12)	36/70	0.97 (0.60, 1.57)	106/107	0.78 (0.56, 1.09)	
Quartile 4	108/160	0.73 (0.54, 0.97)	32/65	0.91 (0.55, 1.51)	76/95	0.64 (0.45, 0.91)	
*P*-trend		0.023		0.686		0.016	
**Soy foods intake (g/d)^e,f^**							
Quartile 1	246/196	1.00 (reference)	61/78	1.00 (reference)	185/118	1.00 (reference)	0.013
Quartile 2	187/218	0.68 (0.51, 0.91)	69/117	0.74 (0.46, 1.19)	137/126	0.70 (0.50, 1.00)	
Quartile 3	169/253	0.65 (0.50, 0.86)	50/92	0.67 (0.40, 1.12)	147/151	0.60 (0.43, 0.85)	
Quartile 4	216/268	0.52 (0.39, 0.69)	34/99	0.43 (0.25, 0.73)	135/154	0.55 (0.39, 0.77)	
*P*-trend		< 0.001		0.003		< 0.001	

*^a^Alternate Chinese Diet Score (aCHD), the soy-free alternate Chinese Diet Score (soy-free aCHD), and soy foods daily intake calculated as quartiles of a linear predictor.*

*^b^OR from logistic regression models adjusted for age at diagnosis for cases or enrollment for controls, area, education, tobacco smoking, moderate physical activity, oral contraceptives use, hormone replacement therapy, family history of breast cancer, history of benign breast disease, age at menarche, number of full-term births, age at first full-term delivery, breastfeeding, and body mass index. Additional adjustment of menopausal age for postmenopausal women.*

*^c^The quartile cut points of aCHD (score) were from control group: Quartile 1 (0–3), Quartile 2 (4), Quartile 3 (5), Quartile 4 (6–9).*

*^d^The quartile cut points of soy-free aCHD (score) were from control group: Quartile 1 (0–2), Quartile 2 (3), Quartile 3 (4), Quartile 4 (5–8).*

*^e^The quartile cut points of soy foods intake (g/d) were from the control group: Quartile 1 (0–3.3), Quartile 2 (3.4–28.6), Quartile 3 (28.6–57.1), Quartile 4 (≥ 57.1).*

*^f^When assessing the independent effect of soy foods, further adjusted for vegetables (g/d), fruit (g/d), nuts (g/d), cereals (g/d), fish (g/d), and monounsaturates (g/d).*

*^g^Diet × Menopause.*

**TABLE 3:** Interaction between the vegetable-fruit-soy diet adherence and the duration on breast cancer risk, stratified by menopausal status.

	Duration < median^a^		Duration≥median^a^		*P* interaction^d^
	Case/Control	Adjusted OR^b^ (95%CI)	Case/Control	Adjusted OR^b^ (95%CI)	
**aCHD (score)**					
Quartile 1	109/153	1.00 (reference)	133/99	1.00 (reference)	0.022
Quartile 2	97/121	1.14 (0.79, 1.65)	136/107	0.95 (0.66, 1.36)	
Quartile 3	82/108	1.11 (0.76, 1.63)	111/103	0.81 (0.55, 1.17)	
Quartile 4	63/104	0.83 (0.55, 1.25)	87/140	0.47 (0.32, 0.68)	
*P*-trend		0.483		<0.001	
**Premenopausal aCHD (score)**					
Quartile 1	19/64	1.00 (reference)	33/42	1.00 (reference)	0.082
Quartile 2	22/53	1.38 (0.68, 2.83)	41/42	1.24 (0.66, 2.33)	
Quartile 3	28/45	2.04 (1.01, 4.10)	30/38	1.03 (0.53, 2.00)	
Quartile 4	13/44	0.97 (0.43, 2.17)	28/58	0.62 (0.33, 1.19)	
*P*-trend		0.578		0.104	
**Postmenopausal aCHD (score)**					
Quartile 1	90/89	1.00 (reference)	100/57	1.00 (reference)	0.013
Quartile 2	75/68	1.08 (0.70, 1.69)	95/65	0.86 (0.54, 1.36)	
Quartile 3	54/63	0.84 (0.52, 1.34)	81/65	0.72 (0.45, 1.16)	
Quartile 4	50/60	0.81 (0.50, 1.31)	59/82	0.42 (0.26, 0.69)	
*P*-trend		0.282		<0.001	

*^a^The duration of the Mediterranean diet intake of premenopausal women and postmenopausal women is calculated separately. The median cut points were from the control group. All subjects: 25 years; premenopausal subjects: 21 years; postmenopausal subjects: 25 years.*

*^b^OR from logistic regression models adjusted for age at diagnosis for cases or enrollment for controls, area, education, tobacco smoking, moderate physical activity, oral contraceptives use, hormone replacement therapy, family history of breast cancer, history of benign breast disease, age at menarche, number of full-term births, age at first full-term delivery, breastfeeding, and body mass index. Additional adjustment of menopausal age for postmenopausal women.*

*^c^The quartile cut points of aCHD were from the control group: Quartile 1 (0–3), Quartile 2 (4), Quartile 3 (5), Quartile 4 (6–9).*

*^d^aCHD × Duration.*

Breast cancer risk in association with the quartile of soy foods intake and the modified soy-free aCHD score is also shown in Table 2. After controlling other types of beneficial foods components in the vegetable-fruit-soy dietary pattern, the consumption of soy foods still showed a stable inverse association with breast cancer among postmenopausal women (4th vs. 1st quartile, OR was 0.55; 95%CI = 0.39, 0.77; *P-*value for trend < 0.001). When all soy foods were excluded from the aCHD score calculation, a high soy-free aCHD score was also found to be inversely associated with postmenopausal breast cancer (OR was 0.64; 95%CI = 0.45, 0.91; *P-*value for trend = 0.016).

For the impact of the separate food components of the vegetable-fruit-soy dietary pattern on breast cancer, we found alcohol consumption to be a significant risk factor for postmenopausal breast cancer (≥median intake vs. < median intake, OR = 3.29; 95%CI = 1.25, 10.3), and a high intake of soy foods was inversely associated with breast cancer (≥median intake vs. <median intake, OR = 0.53; 95%CI = 0.41, 0.70). No significant association was observed between separate food components intake and premenopausal breast cancer risk (Table 4). The average intake of separate foods components of the vegetable-fruit-soy dietary pattern of cases and controls are presented in supplementary material (Supplementary Table 1).

**TABLE 4:** Associations (OR and 95%CIs) between separate foods component of the vegetable-fruit-soy dietary pattern and breast cancer, stratification by menopausal status.

	All		Premenopausal		Postmenopausal	
Dietary variables^b^	Adjusted OR^a^ (95%CI)		Adjusted OR^a^ (95%CI)		Adjusted OR^a^ (95%CI)	
	Intake < median	Intake≥median	Intake < median	Intake≥median	Intake < median	Intake≥median
Coarse cereals (g/d)	1.00 (reference)	0.94 (0.76, 1.17)	1.00 (reference)	1.26 (0.85, 1.87)	1.00 (reference)	0.86 (0.66, 1.13)
Red and processed meat (g/d)	1.00 (reference)	0.96 (0.77, 1.19)	1.00 (reference)	1.19 (0.80, 1.78)	1.00 (reference)	0.87 (0.66, 1.14)
Fish (g/d)	1.00 (reference)	1.05 (0.85, 1.29)	1.00 (reference)	1.16 (0.79, 1.71)	1.00 (reference)	0.99 (0.77, 1.29)
Fruit (g/d)	1.00 (reference)	0.94 (0.75, 1.16)	1.00 (reference)	1.04 (0.70, 1.55)	1.00 (reference)	0.89 (0.68, 1.17)
Vegetables (g/d)	1.00 (reference)	0.98 (0.79, 1.22)	1.00 (reference)	1.01 (0.69, 1.47)	1.00 (reference)	0.95 (0.72, 1.24)
Soy foods (g/d)	1.00 (reference)	0.60 (0.48, 0.74)	1.00 (reference)	0.75 (0.51, 1.10)	1.00 (reference)	0.53 (0.41, 0.70)
Nuts (g/d)	1.00 (reference)	0.88 (0.69, 1.11)	1.00 (reference)	1.04 (0.69, 1.55)	1.00 (reference)	0.81 (0.60, 1.08)
Alcohol (g/d)	1.00 (reference)	1.84 (0.89, 3.95)	1.00 (reference)	0.67 (0.13, 2.55)	1.00 (reference)	3.29 (1.25, 10.3)
MUFA:SFA	1.00 (reference)	0.97 (0.78, 1.22)	1.00 (reference)	1.38 (0.93, 2.04)	1.00 (reference)	0.82 (0.62, 1.09)

*^a^OR from logistic regression models adjusted for age at diagnosis for cases or enrolment for controls, area, education, tobacco smoking, moderate physical activity, oral contraceptives use, hormone replacement therapy, family history of breast cancer, history of benign breast disease, age at menarche, number of full-term births, age at first full-term delivery, breastfeeding, and body mass index. Additional adjustment of menopausal age for postmenopausal women.*

*^b^The median cut points of separate foods component were from the control group. All subjects: coarse cereals (145.28 g/d), red and processed meat (42.86 g/d), fish (28.57 g/d), fruit (103.7 g/d), vegetables (273.4 g/d), soy foods (28.58 g/d), nuts (5 g/d), alcohol (10 g/d), MUFA:SFA (2.6). Premenopausal subjects: coarse cereals (146.52 g/d), red and processed meat (43.44 g/d), fish (28.57 g/d), fruit (105.3 g/d), vegetables (272.3 g/d), soy foods (28.60 g/d), nuts (5 g/d), alcohol (10 g/d), MUFA:SFA (2.6). Postmenopausal subjects: coarse cereals (144.11 g/d), red and processed meat (42.86 g/d), fish (28.57 g/d), fruit (103.7 g/d), vegetables (273.4 g/d), soy foods (28.58 g/d), nuts (5 g/d), alcohol (10 g/d), MUFA:SFA (2.6).*

Since the significant inverse association with the vegetable-fruit-soy diet adherence was only found in postmenopausal breast cancer, further analyses for different breast tumor molecular subtypes were restricted only to the postmenopausal participants. We observed the vegetable-fruit-soy diet adherence was significantly inversely associated with of ER- subtype (4th vs. 1st quartile, OR = 0.63; 95%CI = 0.37, 0.94; *P* trend = 0.003); results were similar for ER-/PR- subtype (4th vs. 1st quartile, OR = 0.64; 95%CI = 0.41, 0.93; *P* trend = 0.012) (Table 5). We did not observe any significant associations between the vegetable-fruit-soy diet adherence and ER+ subtype (4th vs. 1st quartile, OR = 0.84; 95%CI = 0.41, 1.51; *P* trend = 0.371). A test for heterogeneity examines the null hypothesis that the beneficial effect of vegetable-fruit-soy diet adherence on the ER- subtype is the same as that of the ER+ subtype. However, the test of heterogeneity yields a *P*-value of 0.013, conventionally interpreted as being non-significant. Results for ER+/PR+ and ER–/PR– breast cancers were similar to those for ER+ and ER– breast tumors, respectively; results for ER–/PR– /HER2– were not significant.

**TABLE 5:** Associations (OR and 95%CIs) between the vegetable-fruit-soy diet adherence and different breast tumor molecular subtypes in postmenopausal women.^a^

	Quartile 1^b^	Quartile 2^b^	Quartile 3^b^	Quartile 4^b^	*P*-trend
**Total invasive breast cancer**					
Case/Control	190/146	170/133	135/128	109/142	
Age-adjusted	1.00 (reference)	1.05 (0.85, 1.37)	0.95 (0.77, 1.22)	0.68 (0.51, 0.84)	0.002
MV-adjusted^c^	1.00 (reference)	0.97 (0.71, 1.33)	0.78 (0.56, 1.09)	0.57 (0.41, 0.80)	<0.001
**ER+ breast cancer**					
Case/Control	66/146	54/133	53/128	49/142	
Age-adjusted	1.00 (reference)	1.17 (0.82, 1.58)	0.96 (0.43, 1.42)	0.80 (0.45, 1.43)	0.082
MV-adjusted^c^	1.00 (reference)	1.21 (0.70, 1.73)	1.01 (0.39, 1.63)	0.84 (0.41, 1.51)	0.371
**ER– breast cancer**					
Case/Control	32/146	35/133	24/128	25/142	
Age-adjusted	1.00 (reference)	0.98 (0.72, 1.27)	0.84 (0.66, 1.24)	0.75 (0.53, 1.12)	0.023
MV-adjusted^c^	1.00 (reference)	0.92 (0.74, 1.17)	0.72 (0.51, 1.22)	0.63 (0.37, 0.94)	0.003
**ER+/PR+ breast cancer**					
Case/Control	68/146	54/133	54/128	52/142	
Age-adjusted	1.00 (reference)	1.03 (0.66, 1.77)	0.85 (0.55, 1.36)	0.77 (0.58, 1.37)	0.613
MV-adjusted^c^	1.00 (reference)	0.96 (0.62, 1.80)	0.79 (0.51, 1.47)	0.78 (0.53, 1.38)	0.357
**ER–/PR- breast cancer**					
Case/Control	42/146	41/133	33/128	34/42	
Age-adjusted	1.00 (reference)	0.97 (0.66, 1.24)	0.83 (0.65, 1.22)	0.76 (0.56, 1.13)	0.073
MV-adjusted^c^	1.00 (reference)	0.94 (0.73, 1.53)	0.75 (0.49, 1.34)	0.64 (0.41, 0.93)	0.012
**ER–/PR–/HER2– breast cancer**					
Case/Control	10/146	9/133	12/128	5/142	
Age-adjusted	1.00 (reference)	1.08 (0.68, 3.12)	1.14 (0.62, 2.97)	0.67 (0.13, 1.96)	0.428
MV-adjusted^c^	1.00 (reference)	1.45 (0.37, 4.04)	1.52 (0.49, 3.75)	0.65 (0.14, 2.75)	0.511

*^a^We collected the estrogen receptor (ER) information of 439 of 818 cases, including 301 ER-positive and 138 ER-negative.*

*^b^The quartile of the alternate Chinese Diet Score (aCHD), Quartile 1 (0–3), Quartile 2 (4), Quartile 3 (5), Quartile 4 (6–9).*

*^c^Adjusted for age at diagnosis for cases or enrollment for controls, area, education, tobacco smoking, moderate physical activity, oral contraceptives use, hormone replacement therapy, family history of breast cancer, history of benign breast disease, age at menarche, number of full-term births, age at first full-term delivery, breastfeeding, and body mass index. Additional adjustment of menopausal age for postmenopausal women.*

## Discussion

We found that the vegetable-fruit-soy diet adherence was inversely associated with postmenopausal breast cancer risk, especially with ER- and ER-/PR-subtypes. This favorable influence of the vegetable-fruit-soy diet adherence on postmenopausal breast cancer risk cannot be explained by the single effect of soy foods since only a very slight attenuation in estimates was noted after excluding the effect of soy foods. Our finding supports the hypothesis that food components and their combinations in the vegetable-fruit-soy dietary pattern play an etiologic role in breast cancer.

The abundant vegetables and moderate fruits intake are the common features of the Mediterranean and Chinese traditional diets (11). Both diets recommend taking more high-quality white meat rather than red meat (12, 13). However, there are also obvious differences between the two; the main differences are the preference of the two diets in the choice of legumes and oils. Legumes in the traditional Mediterranean diet which includes lentils, peas, beans, chickpeas, and the like; chickpeas (also known as garbanzo beans) and fava beans are two of the most common beans in Mediterranean cooking, but other varieties, such as black beans and kidney beans, can also be seen. On the other side, beans are a typical component in the traditional Chinese diet, which included soybeans, broad beans, peas, mung beans, black beans, and many other varieties, and soybeans and related products are the most common. Another major difference is the choice of oils; the Mediterranean region mainly consumes olive oil while the Chinese use other unsaturated fat cooking oils other than olive oil, such as rapeseed, soybean, flaxseed, peanut, corn, and sunflower oils, and rapeseed oils and soybean oils are the most common. Therefore, the migrated vegetable-fruit-soy diet is not a traditional Mediterranean diet because soybeans and rapeseed oils were not traditional Mediterranean foods. However, both of them could be associated with healthy fat characteristics of the traditional Mediterranean diet, and beans provide fiber, protein, carbohydrate, B vitamins, iron, copper, magnesium, manganese, zinc, and phosphorous. They are naturally low in fat and are practically free of saturated fat, and because they are plant foods, they are cholesterol-free as well. Olives oils provide potential breast cancer benefits, which may because they have a high monounsaturated/saturated fat ratio, while rapeseed oils also have a high polyunsaturated/saturated fat ratio with a similar protective effect to that of monounsaturated oil in reducing breast cancer risk. In terms of grain intake, the Mediterranean inhabitants included whole grains as the main carbohydrate source years ago, as was also used in China before the 1990s (25). The distinction is that the former diet included non-refined barley and wheat, the latter mainly consumes coarse cereals. Although differences in dietary composition between the two regions exist, the main aim of the study is to conclude the main commonalities that are major protective factors of breast cancer.

Many have studied the relationship between individual dietary factors and breast cancer. Alcohol consumption appears to be a convincing risk factor (26, 27), whereas fresh fruit and vegetables appear to be protective (28, 29). However, there is still little evidence for the role of most individual foods or nutrients on breast cancer risk. In this study, alcohol intake was also positively associated with breast cancer, while soy foods consumption seems to be the only food ingredient that was negatively correlated with breast cancer. Soy foods are an important potential beneficial factor in traditional Chinese diet practice and our findings are consistent with previous studies (23, 24). The biological mechanisms for soy isoflavones to reduce the risk of breast cancer may be related to preferential binding to ER-b relative to ER-a (30). Soy isoflavones may also act *via* estrogen-independent mechanisms, such as inhibiting the nuclear transcription factor jB DNA binding activity and the Akt signaling pathway (31), both of which are vital to maintaining a steady-state balance between survival and apoptosis of the cell. Although soy foods seem to be the only food ingredient that may reduce the risk of breast cancer in the present analysis; the favorable influence of the dietary pattern cannot only be explained by the independent effect of separate food ingredients. This is because, when we control the favorable influence of soy foods, the beneficial effects of the vegetable-fruit-soy dietary pattern were still significant.

The possible beneficial effects of the vegetable-fruit-soy dietary pattern on breast cancer can borrow from the physiological mechanisms of the traditional Mediterranean diet. The Mediterranean diet is characterized by a diversity of various polyphenols, especially from nuts, fruits, and legumes, and suggests a potential approach in population to increase polyphenols intake (32). Epidemiological evidence indicates that long-term consumption of a diet rich in plant polyphenols could have a negative impact on the initiation and proliferation of breast cancer, especially with the anti-inflammatory and antioxidant effects that can neutralize free radicals and prevent DNA damage (5, 33–36). Phytoestrogens may have a tumor-inhibiting role as they act like estrogen. The favorable fatty acid profile of the Mediterranean diet may influence breast cancer risk by reducing hyperinsulinemia in cells (3, 16). As fiber intake increases, circulating estrogen and androstenedione levels may decrease, which affects the carcinogenesis of breast cancer (37, 38). The heterogeneity of menopausal status on breast cancer may be due to the greater influence of genetic factors and early-life events of premenopausal breast cancer, which result in the possible protective effect of the vegetable-fruit-soy dietary pattern being more difficult to observe in premenopausal breast cancer (5, 16).

Several prospective studies examined relations between the Mediterranean diet and breast cancer risk, with most (2–6, 8) studies demonstrating an inverse correlation and especially among hormone receptor-negative, but not all (39, 40). The etiology of different breast tumor molecular subtypes may be different, but the evidence remains unclear, especially in different racial populations (40–42). Our finding suggests that the vegetable-fruit-soy dietary pattern is inversely associated with postmenopausal breast cancer and that the inverse association was greater and easier to detect among ER- tumors. As has been suggested before (2, 5), any potential influence of dietary factors may be difficult to detect in ER+ tumors given the strong influence of hormonal factors. In ER- tumors, other risk factors, including diet, may exert a relatively larger influence and may be more easily detectable (2, 16). Findings between different breast tumor molecular subtypes and the vegetable-fruit-soy diet adherence may be particularly important because ER- tumors have a poor prognosis and lower survival rates than ER+ tumors (8, 16, 43).

Several limitations of this study should be noted. First, the data were collected from a case-control study, which might be partially influenced by the selection and recall biases inherent in the design. However, we only included newly diagnosed patients with breast cancer to reduce the recall bias. In addition, the dietary preference was collected based on composite measures, which were less likely to cause selective bias on specific foods/food groups. Second, the vegetable-fruit-soy dietary pattern is not a traditional Mediterranean diet, but the components of the alternative foods of the vegetable-fruit-soy diet that were related to the health characteristics of the traditional Mediterranean diet. The results demonstrated that the foods components and their combinations in the vegetable-fruit-soy dietary pattern may supply benefits in breast cancer prevention and are more suitable for the Chinese population. Finally, the sample size of molecular subtypes of breast tumor are small; we collected the ER information of 439 of 818 cases, including 301 ER-positive and 138 ER-negative. Although there is a suggestion that the Mediterranean diet is inversely associated with ER-/PR-/HER2- (“triple-negative” tumors) (16), our results may lack enough statistical power to find it.

The strengths of our study were the use of a validated food-frequency questionnaire and a modified questionnaire surrounding the Mediterranean dietary pattern and individual dietary factors to examine their association with breast cancer risk in the Chinese female population. Since the diet is a modifiable risk factor, identifying the characterization of the population most susceptible to harmful and beneficial dietary habits is critical for breast cancer prevention policy design. Our findings support vegetable-fruit-soy diet compliance associated with reduced postmenopausal breast cancer risk, especially with ER- or ER-/PR- subtypes.

## Conclusion

The favorable influence from the Mediterranean diet pattern may also apply to Chinese women, and the vegetable-fruit-soy dietary pattern reduced the risk of postmenopausal breast cancer, particularly among ER- subtype, and ER–/PR–subtype.

## Data Availability Statement

The original contributions presented in the study are included in the article/Supplementary Material, further inquiries can be directed to the corresponding author.

## Ethics Statement

The studies involving human participants were reviewed and approved by the Review Boards of the Jiangsu Centers for Disease Control. The patients/participants provided their written informed consent to participate in this study.

## Author Contributions

MW, SC, and PW designed and conducted the study. QZ, ZZ, and JZ developed diet indices and data collection. SC and LL performed the statistical analyses and drafted the manuscript. PW and MW interpreted the data, critically revised the manuscript, and had full responsibility for the analyses and interpretation of the data. SC has full access to all study data. All authors contributed to the preparation of the manuscript and read and approved the final manuscript.

## Conflict of Interest

The authors declare that the research was conducted in the absence of any commercial or financial relationships that could be construed as a potential conflict of interest.

## Publisher’s Note

All claims expressed in this article are solely those of the authors and do not necessarily represent those of their affiliated organizations, or those of the publisher, the editors and the reviewers. Any product that may be evaluated in this article, or claim that may be made by its manufacturer, is not guaranteed or endorsed by the publisher.

## Footnotes

1
http://www.r-project.org/

## Appendix

**APPENDIX A1:** Scoring criteria for alternate Mediterranean diet score and alternate Chinese diet score.

Alternate Mediterranean diet score (18)		Alternate Chinese diet score		Criteria for 1 point^a^
Food group	Foods included	Food group	Foods included	
Whole grains	Whole-grain ready-to-eat cereals, cooked cereals, crackers, dark breads, brown rice, other grains, wheat germ, bran, popcorn	Coarse cereals	Corn, millet, black rice, purple rice, sorghum, barley, oats, buckwheat, other cereals	Greater than median intake (g/d)
Red and processed meat	Hot dogs, deli meat, bacon, hamburger, beef	Red and processed meat	Pork, mutton, beef	Greater than median intake (g/d)
Fish	Fish and shrimp, breaded fish	Fish	Fish, shrimp, shellfish	Greater than median intake (g/d)
Fruit	All fruit and juices	Fruit	All fruit and fresh juices	Greater than median intake (g/d)
Vegetables	All vegetables except potatoes	Vegetables	All vegetables except potatoes	Greater than median intake (g/d)
Legumes	Tofu, string beans, peas, beans	Soy foods	Soybeans, broad beans, peas, mung beans, black beans, other soy foods	Greater than median intake (g/d)
Nuts	Nuts, peanut butter	Nuts	Nuts	Greater than median intake (g/d)
MUFA:SFA	Oliver oil, other monounsaturated oil	MUFA:SFA	Rapeseed oils, other monounsaturated oil	Greater than median intake (g/d)
Alcohol	Wine, beer, “light” beer, liquor		5–25 g/d	5–25 g/d

*^a^0 points if these criteria are not met.*
