# Supplementary material for: Adherence to the Vegetable-Fruit-Soy Dietary Pattern, a Reference From Mediterranean Diet, Protects Against Postmenopausal Breast Cancer Among Chinese Women
Source: Front Nutr. 2022 Mar 29;9:800996. doi: 10.3389/fnut.2022.800996 (PMC9001898; doi:10.3389/fnut.2022.800996)
Supplement: Supplementary file 1 [file Table_1.docx]

Supplementary Material

# Table 1 Means of daily dietary intakes (separate foods component of the vegetable-fruit-soy dietary pattern) among breast cancer cases and controls.

|  | Cases |  | Controls |  |
| --- | --- | --- | --- | --- |
| Dietary variables | Mean |  | Mean |  |
| Vegetables (g/d) | 306.62 |  | 297.38 |  |
| Soy foods (g/d) | 33.30 |  | 44.52 |  |
| Fruit (g/d) | 113.54 |  | 119.36 |  |
| Nuts (g/d) | 13.05 |  | 14.01 |  |
| Cereals (g/d) | 41.78 |  | 40.12 |  |
| Fish (g/d) | 41.16 |  | 37.75 |  |
| MUFA:SFA (g/d) | 0.73 |  | 0.79 |  |
| Red and processed meat (g/d) | 48.69 |  | 52.96 |  |
| Alcohol (g/d) | 2.35 |  | 1.22 |  |
| Energy intake (kcal/d) | 1733.61 |  | 1782.24 |  |

**Mention:** Several studies reported the positive association between high protein-, high fat-containing foods and breast risk in western women is positive^(1)^, due to the diet is rich in saturated fatty acids or trans-monounsaturated fats, which cause pro-inflammatory action^(1)^. However, we did not find a positive correlation between red and processed meat intake and breast cancer risk in this study among Chinese women, may benefit from dietary characteristics of Asian diet similar to the Mediterranean diet, mitigate the low-grade inflammation^(2)^ and regulate the homeostatic balance n-3 PUFAs and n-6 PUFAs. Besides, Chinese women's average red meat intake from our study (19.32 g/1000 kcal/d) was much lower than that among Asian Americans (34.5 g/1000 kcal/d)^(3)^.

Reference:

1. Calder PC, Ahluwalia N, Brouns F *et al.* (2011) Dietary factors and low-grade inflammation in relation to overweight and obesity. *Br J Nutr* 106 Suppl 3, S5-78.

2. Bonaccio M, Pounis G, Cerletti C *et al.* (2017) Mediterranean diet, dietary polyphenols and low grade inflammation: results from the MOLI‐SANI study. 83, 107-113.

3. Wu AH, Yu MC, Tseng CC *et al.* (2009) Dietary patterns and breast cancer risk in Asian American women. *Am J Clin Nutr* 89, 1145-1154.
